# Merging dorsal and ventral striatal pathway outputs of basal ganglia circuit in decision making process

**DOI:** 10.1186/1471-2202-14-S1-P352

**Published:** 2013-07-08

**Authors:** Selin Metin, Neslihan S Sengor

**Affiliations:** 1 Electronics Engineering Department, Istanbul Technical University, Istanbul, 34469, Turkey

## 

Striatum is the critical structure in goal directed behavior. Striatal processing of the input to striatal structures under the presence of dopamine yields the primary output of the action selection loop in the basal ganglia. This primary output travels through the other structures active for the action selection loop in the basal ganglia and produces the stimulation to the motor circuits of the brain via thalamus. It is not wrong to say that action selection starts and ends in the striatum. The recent research shows that both the ventral (nucleus accumbens) and the dorsal (putamen) striatum take part in decision making processes [1]. Midbrain dopamine cells modulate the result of the processing in striatal neurons [2]. Nucleus accumbens, especially shell region, has an important function in determining the reward value of tasks in goal-directed behavior and the error in expectation [2]. This reward value may cause an action to be selected even when the dorsal striatal pathway works against this selection in especially effort-related decision making processes [3].

A computational model is developed to demonstrate that through the striato-nigro-striatal pathway, limbic regions have impact on the motor regions of the basal ganglia and also the integration of the outputs of dorsal and ventral striatal structures produces the resulting output of the basal ganglia action selection loop. This model takes into account the physiological properties of neurons in each basal ganglia structure and the effects of the ion channels on the cell membrane to state a more realistic processing unit. Hodgkin-Huxley type conductance based neuron models are used in order to demonstrate the effects of ion channel currents on the functioning of a neuron. Striatal neurons are modeled as two groups which have D1 or D2 type dopamine receptors. Dopamine input from midbrain neurons acts on the ion channels and stimulates the D1 neurons while inhibiting the D2 neurons. Thus a computational model is obtained which can produce different types of action potentials. This computational model was partially presented at [4] and focuses on the dorsal and ventral striatal pathway interaction under different inputs.
